# *In vitro* and *in vivo* approaches demonstrating the antiproliferative activity of pomegranate seed proteins and peptides on breast cancer

**DOI:** 10.1371/journal.pone.0330895

**Published:** 2025-09-18

**Authors:** Miriam Guzmán-Lorite, Laura Muñoz-Moreno, María Luisa Marina, María José Carmena, María Concepción García

**Affiliations:** 1 Universidad de Alcalá, Departamento de Química Analítica, Química Física e Ingeniería Química, Ctra. Madrid-Barcelona Km. 33.600, 28871 Alcalá de Henares, Madrid, Spain; 2 Universidad de Alcalá, Departamento de Biología de Sistemas, Ctra. Madrid-Barcelona Km. 33.600, 28871 Alcalá de Henares, Madrid, Spain; 3 Universidad de Alcalá, Instituto de Investigación Química “Andrés M. del Río”, Ctra. Madrid-Barcelona Km. 33.600, 28871 Alcalá de Henares, Madrid, Spain; Universidad San Francisco de Quito - Campus Cumbaya: Universidad San Francisco de Quito, ECUADOR

## Abstract

Pomegranate seed proteins and peptides exhibit high antioxidant capacity according to previous work carried out by our group. The present work aims to evaluate the antitumoral potential of pomegranate seed protein isolates (PIs), obtained using two different procedures, pressurized liquids (PLE) and high-intensity focused ultrasounds (HIFU), and their hydrolysates on breast cancer cells (HCC1806) and immunodeficient mice (Hsd:Athymic Nude-*Foxn1*^*nu*^). The treatment of HCC1806 cells with increasing concentrations of pomegranate seed PIs and the hydrolysates significantly reduced cell viability. At a concentration of 4 mg/mL (PLE PI), 2 mg/mL (HIFU PI), and 2.4 mg/mL (hydrolysates), all treatments significantly reduced breast cancer cell migration and metalloproteinase 9 (MMP-9) activity (active and pro form), and enhanced cell adhesion. Additionally, a reduction in the proliferation capacity of HCC1806 cells was observed, most likely due to cell cycle arrest and the apoptotic effect of PLE PI and its hydrolysate. *In vivo* experiments with pomegranate seed hydrolysates confirmed their antitumoral capacity, observing significant reductions in the tumor volume and weight after treating with pomegranate proteins and peptides, as well as in metalloproteinase (MMP) activity and vascular endothelial growth factor (VEGF) expression. In addition to proteins and peptides, the anticancer potential may also be related to the presence of phenolic compounds, especially abundant in the PLE extracts.

## 1. Introduction

Breast cancer is the most frequent cancer type in women worldwide, accounting for 2.3 million women diagnosed in 2022, and 670,000 deaths caused in the same year, according to the World Health Organization [1]. The current tendency for this disease is predicted to increase to over 3 million new cases and 1 million deaths every year by 2040 [2]. This scenario reveals the need for new preventive tools and treatments for the disease.

Pomegranate (*Punica granatum* L.) is composed of peels, arils (edible part), and seeds. This fruit and extracts from different parts of the fruit have been associated with multiple health benefits, including effects on inflammatory, neoplastic, cardiovascular, metabolic, and viral diseases, among others [3]. The phenolic compounds contained in pomegranate are considered the primary contributors to these beneficial properties, although some fatty acids present in the seeds, such as punicic acid, have also exhibited interesting bioactivities [4]. Pomegranate extracts have already been reported to possess anticancer capacity by modulating various cancer-related processes such as cellular proliferation, cell cycle, inflammation, or angiogenesis [5,6]. Several studies have also specifically explored their potential against breast cancer, reporting the modulation of key hormones involved in this disease [7].

Although phenolics and fatty acids from pomegranate have been widely studied, the protein fraction of the fruit, which accounts for approximately 20% of the seed, has received limited attention. In previous studies, our group demonstrated the antioxidant, antihypertensive, and hypocholesterolemic potential of pomegranate peel proteins and peptide-enriched extracts [8]. Moreover, pomegranate seed protein extracts and their hydrolysates also demonstrated a significant antioxidant capacity [9,10]. In addition to proteins and peptides, these extracts also contained phenolic compounds and other substances, which contributed to their functionality.

Considering these antioxidant properties and the link between the overproduction of reactive oxygen species (ROS) and cancer development [3,11], the objective of this work was to go further in this line by evaluating the effect of pomegranate seed protein and peptide extracts on breast cancer. For that, two pomegranate seed protein isolates (PIs), obtained by two different procedures (high-intensity focused ultrasounds (HIFU) and pressurized liquids (PLE)), and their corresponding hydrolysates were compared for their ability to treat breast cancer cells (HCC1806) in *in vitro* and *in vivo* studies. Cell viability, wound healing capability, adhesion, cell cycle, proliferation, tumor growth, and metalloproteinases (MMPs) activity were evaluated and results from all four samples were compared. *In vivo* experiments were carried out using immunodeficient mice (Hsd:Athymic Nude-*Foxn1*^*nu*^). After tumor induction using HCC1806 cells previously treated with the pomegranate seed extracts, tumor volume and weight were measured, and vascular endothelial growth factor (VEGF) expression and MMPs activity were assessed.

## 2. Materials and methods

### 2.1 Materials

Roswell Park Memorial Institute (RPMI-1640), keratinocyte serum-free medium (K-SFM), Matrigel matrix, phosphate buffer saline (PBS), dimethyl sulfoxide (DMSO), trypsin, glacial acetic acid, methanol, and ethanol were all purchased from Fischer Scientific (Geel, Belgium). Gelatin, Coomassie Brilliant Blue R‐250, 3-(4,5-dimethylthiazol-2-yl)-2,5-diphenyltetrazolium bromide (MTT), sodium dodecyl sulfate (SDS), tris-aminomethane (Tris), bovine serum albumin (BSA), Triton X-100, collagen, calcium chloride (CaCl_2_), sodium chloride (NaCl), sodium azide, nonidet P40, ortovanadate, aprotinin, leupeptin, and pepstatin. were obtained from Sigma (Saint-Louis, MO, USA). Fetal bovine serum (FBS) and antimycotic/antibiotic (penicillin/streptomycin/amphotericin B) were acquired from Life Technologies (Carlsbad, CA, USA). Bovine pituitary and epidermal growth factor were from Gibco (Kandel, Germany). Ethylenediaminetetraacetic acid (EDTA) was from Merck (Darmstadt, Germany). Defatted pomegranate seeds employed for these studies were provided by AMC Innova Juice and Drinks, S.L (Murcia, Spain).

### 2.2 Preparation of pomegranate seed protein isolates and hydrolysates

The seeds employed in this work were kindly donated by AMC Natural Drinks (Murcia, España) and were obtained from pomegranates harvested in autumn. Protein-enriched pomegranate seed extracts were obtained using HIFU and PLE, following protocols previously described by Guzmán-Lorite et al. (2022) [9]. Peptide-enriched extracts were obtained after the hydrolysis of PIs using pepsin and pancreatin enzymes. The complete procedure was described in Guzmán-Lorite et al. (2022) [9].

### 2.3 Cell culture

Tumoral breast cancer cells from the HCC1806 cell line (American Type Culture Collection, ATCC) were maintained in RPMI-1640 medium supplemented with 10% FBS and 1% antibiotic/antimycotic. The culture was carried out in a humidified 5% CO_2_ environment at 37°C. Cells were washed with PBS, detached with trypsin (0.25%)/EDTA (0.2%), and seeded at 30,000–40,000 cells/mL after reaching 70–80% confluence.

### 2.4 *In vitro* assays

Concentrations of pomegranate seed proteins and peptides employed in all assays were: 4 mg/mL for the PLE PI, 2 mg/mL for the HIFU PI, and 2.4 mg/mL for the peptides. These concentrations were chosen based on the viability assay (section 3.1).

#### 2.4.1 Cell viability.

The MTT assay was used to assess the effect of pomegranate seed PIs and their hydrolysates over the viability of the HCC1806 cells. Following Muñoz-Moreno et al. (2016) protocol, a concentration of 2.5 x 10^5^ cells was seeded in 96-well plates for 24 h. After that time, different concentrations of pomegranate seed PIs and hydrolysates, ranging from 0.1 to 5 mg/mL, were employed for the treatment of cells, whereas non-treated cells were used as control. After another 24 h, cells were incubated for 1.5 h with MTT (5 mg/mL) and after removing the culture medium, the resulting formazan crystals were solubilized in 50 μL DMSO for absorbance measurement (570 nm wavelength) using a Multiskan FC plate reader (Thermo Scientific, Waltham, Massachusetts, USA) [12]. Cell viability was expressed as percentage related to control cells.

#### 2.4.2 Wound healing assay.

Migration capacity of HCC1806 cells treated with PIs and hydrolysates was evaluated by the wound healing assay. This assay involves creating a scratch in a cell monolayer and comparing the wound width at different times and was performed as indicated by Muñoz-Moreno et al. (2018) [13]. Briefly, 24-well plates were employed for cell growth up to confluence. Then, a scratch was performed with a scraper, and cells were cultured in the presence of the proteins and peptides, previously diluted in complete RPMI-1640 medium. A control experiment without any extract was also conducted. The wound healing process was monitored using a Nikon Diaphot 300 inverted microscope (Nikon Instruments Inc., Melvilla, NY, USA) and images were collected at different times (0-24h) after the treatment. The average wound width at different times was calculated, and results were expressed as percentage related to the average wound width at 0h (100%).

#### 2.4.3 Evaluation of cell adhesion.

The evaluation of cell adhesion was performed according to Muñoz et al. (2018) [13]. Briefly, HCC1806 cells were collected by the addition of 0.25% trypsin/0.2% EDTA. The suspension was immediately centrifuged, and the pellet was resuspended in culture medium containing 0.1% (w/v) BSA (pH 7.4) and treated with pomegranate seed proteins and peptides. After that, cells were added to an artificial collagen matrix at a concentration of 2.5 x 10^5^ cells/mL. This collagen matrix was previously formed using a type-I collagen solution, that was prepared in 10 mM glacial acetic acid and incubated in 96 well-plate (37°C, 1 h), followed by washing twice with PBS (pH 7.4). After incubating the cells in the collagen matrix for 60 minutes, cell adhesion was quantified by treating the samples with 1 mg/mL MTT for 1 h at 37°C and 5% CO₂. The resulting formazan precipitate was then dissolved in DMSO, and absorbance was measured at a wavelength of 570 nm. Control cells (non-treated) were assigned a value of 100%, and the results were expressed as a percentage relative to the control.

#### 2.4.4 Gelatin zymography.

Activity of MMP‐2 and MMP‐9 in HCC1806 cells was determined by gelatin zymography [14]. For that, HCC1806 cells were seeded at a concentration of 1.5 x 10^4^ cells/well in 6 well-plates and then incubated (37°C, 5% CO_2_) for 24 h. After that, culture medium was removed, and cells were grown in conditioned media stimulated with pomegranate seed samples for another 24 h. Treated medium was collected and analyzed by zymography in 10% SDS-PAGE gels containing 0.1% (w/v) gelatin as substrate. The amount of protein loaded into wells was 3 μg and electrophoresis was carried out at 4°C. After completion, gels were washed twice with 50 mM Tris (pH 7.4) containing 2.5% (v/v) Triton X − 100 for 1 h, followed by two 10‐min rinses in 50 mM Tris (pH 7.4). Following SDS removal, gels were incubated overnight under constant agitation at 37°C in 50 mM Tris (pH 7.5) containing 10 mM CaCl_2_, 0.15 M NaCl, 0.1% (v/v) Triton X‐100, and 0.02% sodium azide. After that, they were stained using Coomassie Brilliant Blue R‐250 (0.25%) and washed with 7.5% acetic acid in 20% methanol. The activity of MMP‐2 and MMP‐9 inactive and active forms was semi-quantitatively determined by densitometry.

The same procedure as described above was employed for the determination of MMPs activity in mice tumor lysates (see section 2.5).

#### 2.4.5 Cell cycle analysis.

To study the effect of pomegranate seed proteins and peptides over the cell cycle, the methodology described by Laura-Muñoz et al. (2016) was followed [12]. HCC1806 cells were seeded in 6 well-plates for 24 h in a complete culture medium (10% FBS and 1% penicillin/streptomycin/amphotericin B). Then, the medium was replaced by RPMI-1640 without FBS, and incubated for another 16 h. Afterwards, cells were treated with pomegranate seed proteins and peptides, washed with PBS, and separated using 0.25% trypsin/0.2% EDTA. The pellet was centrifuged (5 min, 500 *x g*, 4°C) and maintained in ice-cold 70% ethanol for 30 min at −20°C. After centrifugation, the ethanol phase was removed, and the pellet was washed with PBS. The pellet was next analyzed by flow cytometry using a MACSQuant® Analyzer 10 Flow Cytometer (Miltenyi Biotech, 9 Bergisch Gladbach, Germany). Samples were diluted in PBS containing 0.2 mg/mL RNase A and 50 μg/mL propidium iodide. Results were analyzed with the MACSQuantify v 2.13.1 program (Miltenyi Biotech, 9 Bergisch Gladbach, Germany).

#### 2.4.6 Clonogenic assay.

To estimate the effect of pomegranate seed PIs and their hydrolysates over the proliferation of breast cancer cells, a clonogenic assay was carried out according to a previous protocol [15]. Briefly, cells were seeded in 6 well-plates at a density of 100 cells/well in complete medium (1% antibiotic/antimycotic and 10% FBS) and incubated for 24 h. After that, the culture medium was removed, and cells were treated with pomegranate seed extracts for 2 weeks (medium was changed every 48 h). Cells were then attached to the wells using a solution of methanol/acetic acid (3:1) for 30 min, previous culture medium removing. Finally, they were washed with PBS and dyed with 0.5% crystal violet in water/methanol (4:1) for 4 h. Colony formation was quantified by optical density after dissolving formed crystals in methanol. The average clonogenicity of non-treated cells (control samples) was set to 100%, and the colony-forming ability (clonogenicity) of treated cells was expressed as a percentage relative to the control.

### 2.5 Animals, xenografts, and processing of tumors

4 weeks-old female Hsd:Athymic Nude-*Foxn1*^*nu*^ mice were obtained from Inotiv (IN, USA) and maintained in microisolator units feeding a standard diet (standard pellets with 14.3% protein, 48% carbohydrate, and 3.7% fat). The initial weight of the mice was 24.7 ± 0.4 g. Temperature and humidity conditions were controlled, and the light/dark cycle was set at 12 h intervals. All experiments were conducted in compliance with current regulations, particularly Law 32/2002 of November 7, concerning the care of animals during farming, transport, experimentation, and slaughter, as amended by Law 6/2013 of June 11, and by Royal Decree 53/2013 of February 1, which establishes the basic rules for the protection of animals used in scientific procedures. Additionally, all procedures adhered to European Directive 86/609/EEC and the Council of Europe’s European Convention for the Protection of Vertebrate Animals Used for Experimental and Other Scientific Purposes (ETS No. 123). The protocol was approved by Comunidad de Madrid (Spain) (Ref. PROEX 340.6/23). All animal handling was performed by qualified personnel following standardized procedures designed to minimize pain and distress. For that, Meloxicam (2 mg/kg) was administered in drinking water daily for 3 days.

Before the preparation of xenografts, HCC1806 cells were treated with pomegranate seed hydrolysates (2.4 mg/mL) for 24 h. Control cells were non-treated. After that time, cells were detached from flasks using 0.25% trypsin/0.2% EDTA, centrifuged (4000 *x g*, 4°C), and resuspended in fresh complete RPMI medium. To obtain xenografts, the cell suspension was mixed with Matrigel, a synthetic basement membrane (1:1, v/v). Next, animals were randomly assigned to three groups: group 1 (12 mice, control group), group 2 (12 mice, with HIFU hydrolysate-treated cells), group 3 (12 mice, with PLE hydrolysate-treated cells). Xenografts were injected subcutaneously into the right flank of mice (5 x 10^6^ cells/mouse) to form intradermal tumors. Tumor volume (length x width x height x 0.5236) and weight were measured twice a week. All mice were euthanized on day 40 of the experiment with an excess of CO_2_. After that, tumors were dissected, and tumor lysates were homogenized in 1 M Tris-HCl (pH 7.6) containing 1% Nonidet P40, 150 mM NaCl, 2 mM ortovanadate, 5 μg/mL aprotinin, 5 μg/mL leupeptin, and 5 μg/mL pepstatin. The mixture was gently agitated for 30 min in a cold room, and cleared by centrifugation for 30 min at 15000 *x g* and 4°C.

### 2.6 Determination of vascular endothelial growth factor (VEGF)_165_

VEGF levels were estimated in tumor lysates by human VEGF-165 ELISA Development Kit (PeproTech, Kandel, Germany) following manufacturer’s instructions. Data were normalized by the protein concentration in each sample.

### 2.7 Data analysis

Data obtained were analyzed using GraphPad Prism software (GraphPad Software v. 8.0.2, San Diego, CA). Each experiment was carried out at least twice, with 3 replicates of each sample in every replicate. For comparison purposes, analysis of variance (ANOVA) was carried out with a level of significance of 0.05. Data are presented as mean ± standard error of the mean (SEM); *p* < 0.05 was considered statistically significant.

## 3. Results

### 3.1 Effect of pomegranate seed PIs and their hydrolysates over breast cancer cells viability

**Fig 1:**
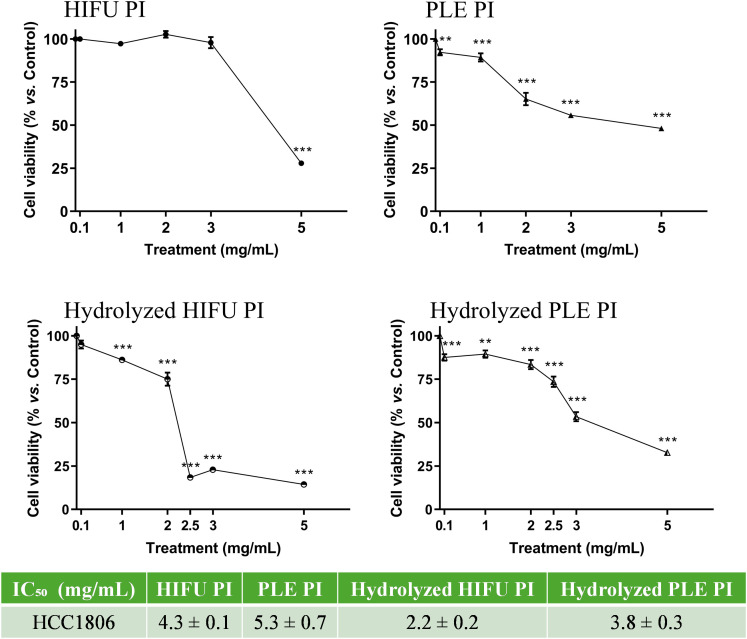
Viability of HCC1806 cells after the treatment with pomegranate seed PIs, obtained by HIFU and PLE, and their hydrolysates at different concentrations (0.1–5 mg/mL). Data are presented as mean ± SEM of three independent experiments; **p < 0.01; ***p < 0.001. IC_50_ values (mg/mL) were calculated for each pomegranate seed treatment.

The viability of HCC1806 breast cancer cells in the presence of pomegranate seed PIs and hydrolysates at concentrations ranging from 0.1 to 5 mg/mL was estimated and results are presented in Fig 1. The treatment of HCC1806 cells with PLE PI and its hydrolysate at concentrations above 0.1 mg/mL resulted in a significant reduction in cell viability, whereas HIFU PI and its hydrolysate required higher concentrations to achieve a similar effect (1 mg/mL for the hydrolyzed HIFU PI and 5 mg/mL for the HIFU PI). The samples with the lowest IC_50_ values, and thus the most effective in reducing cell viability, were the hydrolysates from HIFU and PLE PIs (see Fig 1).

Based on the calculated IC_50_ values, concentrations of 4 mg/mL for PIs and 2.4 mg/mL for hydrolysates were initially selected for subsequent studies. Nevertheless, treatment of normal RWPE-1 cells with 4 mg/mL HIFU PI resulted in cytotoxicity (30 ± 5% cell viability, compared to untreated control cells) and, therefore, this concentration was reduced to 2 mg/mL. At the concentrations chosen, the viability of normal cells varied between 90 ± 2% and 100 ± 10%.

### 3.2 Effect of pomegranate seed PIs and their hydrolysates over cell migration, cell adhesion, and metalloproteinases activity

**Fig 2:**
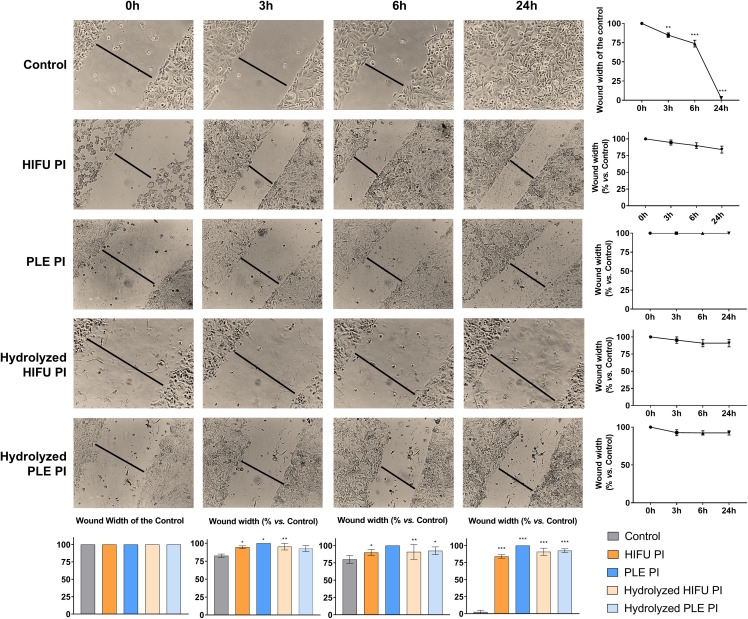
Microscope images of HCC1806 cells after 0, 3, 6, and 24 h of scratching and treating with pomegranate seed PIs (4 mg/mL for that obtained by PLE and 2 mg/mL for the obtained by HIFU) and their corresponding hydrolysates (2.4 mg/mL). Untreated cells served as the control. Wound width was compared across different time points and treatments to assess the effect of samples on the migration capacity. Data are presented as mean ± SEM from three independent experiments; *p < 0.05; **p < 0.01; ***p < 0.001.

The results obtained from the evaluation of tumor cell migration in the presence of pomegranate seed PIs and hydrolysates are shown in Fig 2 in comparison with an untreated control. While tumor cells in the control experiment showed complete wound closure after 24 h, the wounds remained open in the treated cells. A significant reduction in cell migration was achieved with pomegranate seed extracts and hydrolysates as early as 3 hours treatment, with no significant differences between treatments at later time points. The reduction in cell migration, calculated as the difference in wound width between control and treated cells after 24 h of treatment with pomegranate seed PIs and hydrolysates, ranged between 81%, for the HIFU PI, to 89%, for hydrolyzed PLE PI.

**Fig 3:**
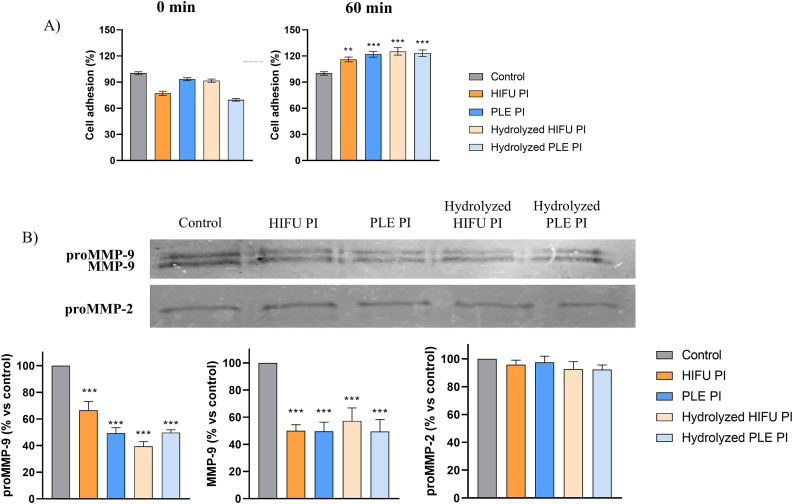
(A) Percentage of cell adhesion at 0 min and after 60 min of treatment with the different pomegranate seed samples. Control (untreated cells) is considered to represent 100% adhesion. (B) Activity levels of MMPs (proMMP-9, proMMP-2, and MMP-9) before (control) and after treatment with pomegranate seed extracts. Data are presented as mean ± SEM of three independent experiments; **p < 0.01; ***p < 0.001. Concentration of treatments: 4 mg/mL for PLE PI; 2 mg/mL for HIFU PI; and 2.4 mg/mL for the hydrolysates.

The effect of the samples on cell adhesion is shown in Fig 3A, along with the untreated control. A significant increase in cell adhesion was observed after 60 min of treatment with all pomegranate seed samples.

Additionally, Fig 3B shows the MMPs activity with and without treatment with pomegranate seed samples. The results demonstrated a significant reduction (45−65%) in MMP-9 and proMMP-9 activity in breast cancer cells treated with all extracts, with no significant differences observed among treated samples. However, no changes were detected in the proMMP-2 activity. Overall, both pomegranate seed PIs and their hydrolysates demonstrated a significant effect on cell migration capacity, adhesion, and MMPs activity.

### 3.3 Effect of pomegranate seed PIs and their hydrolysates over the cell cycle and the proliferation rate

**Fig 4:**
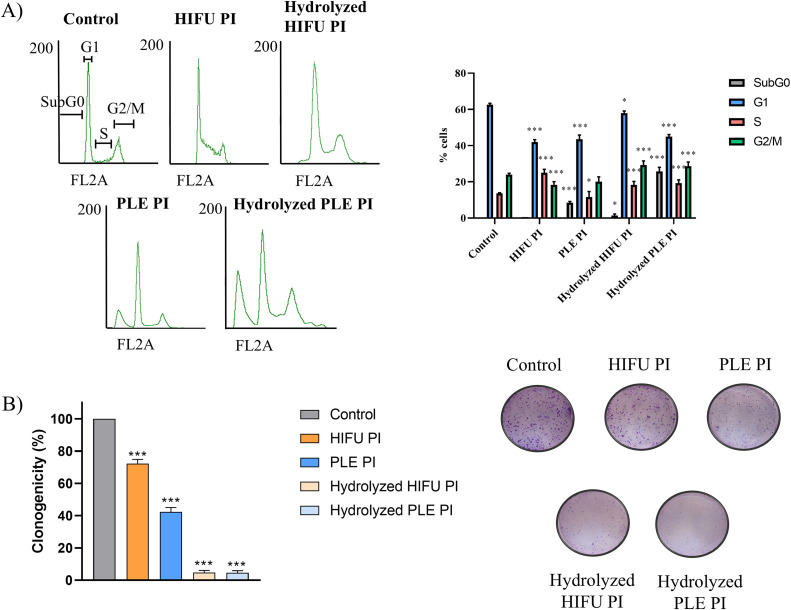
(A) Cell cycle distribution and percentage of cells in each phase before (control) and after treatment of HCC1806 cells with pomegranate seed PIs and hydrolysates.(B) Representative images of HCC1806 cell colonies before (control) and after treatment with pomegranate seed samples. Clonogenicity is expressed as percentage relative to the control (set at 100%). Data are presented as mean ± SEM of three independent experiments; *p < 0.05; ***p < 0.001. Concentration of treatments: 4 mg/mL 4 mg/mL for PLE PI; 2 mg/mL for HIFU PI; and 2.4 mg/mL for the hydrolysates.

Fig 4A shows the effect of pomegranate seed PIs and their hydrolysates on the cell cycle. Treatment with HIFU PI and both hydrolysates resulted in significant increases in the number of cells in the S phase, while HIFU and PLE PIs and the PLE PI hydrolysate resulted in a reduction in the number of cells in the G1 phase. Additionally, the treatment with hydrolysates significantly increases the number of cells in the M phase, and the PLE PI and its hydrolysate induced a marked apoptotic effect (see signal corresponding to SubG0). This effect was even more pronounced with the hydrolysate than with the PI.

The capacity of HCC1806 cells to form colonies in presence of pomegranate seed extracts/hydrolysates is shown in Fig 4B. Proliferation was significantly reduced by all samples, with the hydrolysates showing the strongest effect (96–97% inhibition of proliferation). These results are consistent with those obtained in the cell cycle analysis, in which the hydrolyzed samples, particularly the pomegranate seed PLE hydrolysate, exhibited the highest levels of apoptosis and cell cycle arrest.

### 3.4 *In vivo* confirmation of antitumor activity of pomegranate seed PIs and hydrolysates on HCC1806 cells

**Fig 5:**
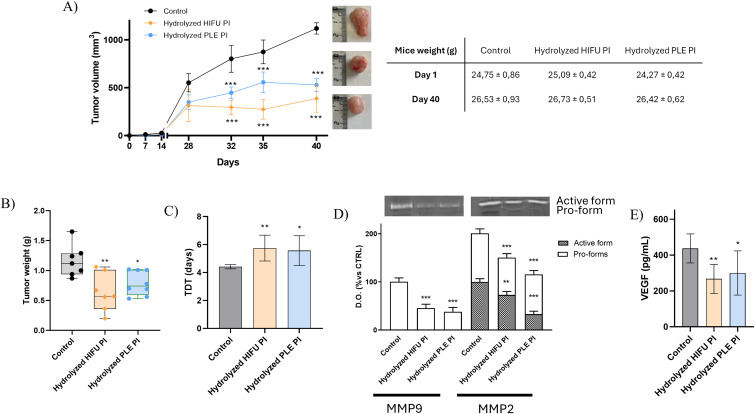
(A) Tumor volume, (B) tumor weight, and (C) TDT, along with (D) MMP activity and (E) VEGF expression, measured in breast tumors from immunodeficient mice previously implanted with either untreated (control) or pomegranate seed hydrolysate-treated HCC1806 cells. Data are presented as mean ± SEM of three independent experiments; *p < 0.05; **p < 0.01; ***p < 0.001. Concentration of treatments: 2.4 mg/mL.

To confirm the results obtained from the *in vitro* assays, breast cancer cells were employed to form intradermal tumors in Hsd:Athymic Nude-*Foxn1*^*nu*^ mice. Only pomegranate seed hydrolysates were used, as they demonstrated higher antitumor capacity than PIs in our previous studies. Non-treated cells were used as control. The results from these *in vivo* experiments are shown in Fig 5. Tumor volume significantly decreased after 32 days in mice treated with pomegranate seed hydrolysates (295 ± 70 mm^3^ for HIFU hydrolysates and 448 ± 62 mm^3^ for PLE hydrolysates), compared to the control mice (803 ± 142 mm^3^). Similarly, tumor weight significantly decreased from 1.2 ± 0.1 g (control) to 0.8 ± 0.07 and 0.6 ± 0.1 g in mice treated with PLE and HIFU hydrolysates, respectively, after 40 days. In correlation with this, tumor doubling time (TDT) increased by 26–30% after the treatment with PLE and HIFU hydrolysates.

Additionally, MMPs activity and the expression of VEGF were assessed in the tumors. VEGF is a protein with a crucial role in the formation of blood vessels and, thus, the growth of tumor cells. Although no effect was observed on the activity of proMMP-2 after *in vitro* treatment with HIFU and PLE hydrolysates, Fig 5D shows a significant reduction in both MMP-2 and MMP-9 activity (active and pro-forms) after treatment with HIFU and PLE hydrolyzed PIs. This reduction was more pronounced following treatment with the PLE hydrolysate.

Both hydrolysates also significantly reduced VEGF expression to 31% (PLE) and 39% (HIFU). The inhibition of VEGF overexpression after treatment with pomegranate seed hydrolysates is consistent with the observed reduction in the tumor volume and weight.

## 4. Discussion

Pomegranate seed protein extracts have demonstrated antioxidant properties based on *in vitro* assays performed in a previous study by the group [9]. This study also showed that the procedure used to extract pomegranate seed proteins (HIFU or PLE) significantly affected the composition of the extracts and their antioxidant properties. A further investigation into the effect of pomegranate seed hydrolysates led to an increase in antioxidant properties in some cases and a decrease in others. Proteins, peptides, and likely co-extracted phenolic compounds seemed to contribute to the overall *in vitro* antioxidant effect. To confirm these effects, these extracts and their hydrolysates have been employed to evaluate their antitumor activity on breast cancer. Specifically, cell viability, migration, adhesion, MMP activity, proliferation rate, and cell cycle progression were assessed in the presence of each extract and hydrolysate. Furthermore, *in vivo* assays have been conducted to validate results.

Cell viability was significantly reduced using treatments from 0.1 mg/mL PLE PI and its corresponding hydrolysate. However, concentrations of 1 mg/mL and 5 mg/mL were necessary to achieve this effect when treating with HIFU samples. These results agree with the antioxidant activity shown by these extracts in *in vitro* assays carried out by our group [9]. Indeed, PLE PI and its hydrolysate exhibited the highest ABTS scavenging capacity (28 ± 5% and 55 ± 4%, respectively) compared to HIFU samples (10 ± 1% for the hydrolysate). This can be linked to the higher presence of more proteins (27 ± 2 g protein/100 g seed for PLE PI *vs*. 14.2 ± 0.7 g protein/100 g seed for HIFU PI) and peptides (degree of protein hydrolysis of 70 ± 6% and 39 ± 8%, respectively) in the PLE samples compared to the HIFU [9]. The antioxidant and antitumoral properties of intact proteins and hydrolysates have been previously connected, showing a significant correlation between both [16]. Additionally, PLE also extracted more phenolic compounds than HIFU: 14.2 ± 0.2 mg GAE/g seed for PLE samples *vs*. 3.1 ± 0.2 mg GAE/g seed for HIFU samples [9,17]. The presence of phenolic compounds has been associated with anticancer bioactivity [18]. In fact, flavonoids and polyphenols, well-known antioxidant molecules, have been reported to induce the expression of various tumor suppressor genes through epigenetic modifications [19]. More specifically, polyphenols present in pomegranate extracts have shown anticancer properties, particularly in breast cancer [20]. Ferulic acid and ellagic acid, two phenolic compounds present in PIs [9], have already been reported to have antitumor activity [21,22].

Consequently, the higher content in proteins, peptides, and phenolic compounds in the PLE PI may be responsible for the differences observed in the reduction of HCC1806 cells viability compared to the HIFU extracts. This dual antioxidant-anticancer activity has been also observed with alperujo pectin–polyphenol complexes, palm fruit pollen polyphenolic extracts, and *Zingiber cassumunar* (*Z. cassumunar*) rhizome protein hydrolysates. In these cases, extracts demonstrated high radical scavenging capacity, cell-based ROS reduction, and antiproliferative activity in colon cancer cells (in the case of the alperujo, palm fruit pollen, Zingiber peptides), and in hepatoma and breast cancer cells (in the case of the palm fruit pollen) [23–25].

IC_50_ values obtained in the treatments of HCC1806 cells with pomegranate seed extracts were comparable to those of the hexapeptide FIMGPY, isolated from skate (Raja porosa) cartilage, which showed an IC₅₀ of 4.81 mg/mL in HeLa cells [26]. However, it is common that IC_50_ values of specific isolated or purified bioactive peptides were lower than those for complex extracts. This is the case of peptide LLPSY from olive seeds, which has an IC_50_ lower than 100 µg/mL in prostate and breast cancer cells [27]. The concentrations of pomegranate seed PIs and their hydrolysates employed for the treatment of HCC1806 cells were based on the viability assay. However, the initial concentration chosen for HIFU PI (4 mg/mL) resulted in cytotoxicity. Consequently, it was reduced to 2 mg/mL*.* HIFU PI did not have a significant effect on HCC1806 viability at a concentration of 2 mg/mL, compared to untreated control cells (see Fig 1). However, a compound or an extract may have non-cytotoxic but cytostatic or anti-metastatic effects, affecting critical tumor functions without inducing direct cell death [28].

Additionally, different processes related to metastasis were also studied. The metastatic process can lead to the development of secondary tumors through the loss of cell-to-cell and cell-to-extracellular matrix (ECM) adhesion, among other factors [29]. In this context, this work evaluated the effect of pomegranate seed PIs and their hydrolysates on HCC1806 cell migration, adhesion, and MMPs activity. The higher migration rate of cancerous cells results in faster wound closure, compared to normal cells. Additionally, normal tissues are structurally maintained through cell-to-cell and cell-to-matrix interactions. In cancerous tissues, these interactions become weaker, leading to reduced cell adhesion and progression to a metastatic stage. Furthermore, the overexpression of enzymes, such as MMPs, that degrade ECM, also contributes to the metastatic process [30]. All pomegranate seed treatments meaningfully reduced cell migration rates and increased cell adhesion. The decrease in migration rates was comparable to the observed with amaranth seed protein hydrolysates, and a similar increase in cell adhesion was reported after the treatment of MDA-MB-468 breast cancer cells with LLPSY from olive seed (37% increase after 80 min) [27,31]. Nevertheless, the treatment with pomegranate seed samples resulted only in the inhibition of MMP-9 and its pro- form but did not affect MMP-2. The activity of MMP-9 is regulated by p28 phosphorylation and subsequent AP-1 activation, whereas TNF-α and the transcription factor NF-κB regulate MMP-2 [32]. Thus, it is possible that the tested pomegranate seed samples exert their effect on MMP-9 signaling cascade but not on MMP-2 activation pathway. Similar results were obtained for the inhibition of MMP-9 and MMP-2 by various legumes; only albumin fraction of *Lupinus albus* (*L. albus)* was able to reduce MMP-2 activity, whereas the rest of the legumes selectively inhibited MMP-9 [33].

The cell cycle constitutes an extensively regulated and coordinated process in which cells divide and duplicate, passing down their genetic information to daughter cells. It is divided into four phases: the G1 phase (cell growth), the S phase (DNA synthesis), the G2/M phase (cell growth and mitosis), and the interphase or SubG0 phase (resting phase, which could result in apoptosis) [27,34]. Alterations in the cell cycle during carcinogenesis lead to aberrant cell proliferation and genetic instability [35]. The treatment with pomegranate seed proteins and peptides resulted in cell cycle arrest: in the S phase with HIFU PI and PLE and HIFU hydrolysates, and in the G2/M phase with the hydrolysates. The inhibition of the cell cycle progression in cancer cell lines occurs with many natural extracts [36]. Moreover, the apoptotic effect observed after treatment with PLE PI, which dramatically raised up with the PLE hydrolysates, may suggest that peptides contribute more to the apoptotic activity than intact proteins. Indeed, peptides have been used in cancer therapy, and specifically, some of them have been classified as apoptotic compounds. There is even a database that collects these peptides [37]. The mechanism of action of anticancer peptides is well-established. Most peptides depend heavily on cationic, amphipathic structures targeting negatively charged cancer cell membranes, leading to permeabilization/lysis and sometimes apoptosis. Others enter cells to disrupt intracellular targets or induce caspase-dependent apoptosis [38,39]. In the case of proteins, its larger size may hinder these interactions, decreasing their anticancer capacity. The blockage of the cell cycle in any of its phases has been studied with natural extracts from American cranberry or *Desmodium gangeticum (D. gangeticum)*. These effects were due to changes in the expression of the proteins regulating the cycle, such as cyclins, p21, p27, etc. [40,41]. The observed reduction in cell proliferation is a consequence of these changes in the cell cycle.

Finally, i*n vivo* experiments confirmed all these *in vitro* results. Similarly, significant reductions in tumor volume and weight were also observed following treatment with other natural extracts, such as a 99% ethanolic extract of *Chamaecyparis obtusa (C. obtusa)* leaves, which effectively reduced breast tumors *in vivo* [42]. VEGF expression was reduced in the tumors treated with all pomegranate seed hydrolysates. Similarly, the treatment of Caco2- and EA.hy926 cells with sardine protein hydrolysates < 10 kDa, at a concentration of 2 mg/mL, also resulted in a significant decrease in VEGF expression [43]. MMPs activity was also reduced by both PLE and HIFU hydrolysates. However, no reduction in the *in vitro* activity of proMMP-2 was observed (Fig 3). The absence of biokinetics processes in the *in vitro* methods may lead to misinterpretation of the data [44]. Moreover, the time of exposition to the treatment also differed significantly between *in vitro* and *in vivo* experiments (hours in the case of *in vitro* experiments vs. days for the *in vivo* experiments), which may further explain this discrepancy.

Further studies are needed to explore the potential applications and implications of pomegranate seed protein extracts and hydrolysates in anticancer therapy. However, current findings with a peptide obtained from pomegranate seeds (PG2) are promising since this peptide has shown a significant capacity to reduce the viability of NB4 and MOLT-4 leukemia cells. Moreover, PG2 exhibited synergistic cytotoxic effects when combined with the anticancer drug daunorubicin, resulting in enhanced therapeutic efficacy [45].

## 5. Conclusions

Multicomponent extracts from pomegranate seeds, containing proteins, peptides, and phenolic compounds, obtained by HIFU and PLE, exerted strong antitumoral activity in breast cancer cells and in immunodeficient mice. PIs and their hydrolysates significantly reduce cell viability, observing the highest effects with the PLE PI and the hydrolyzed HIFU PI. All extracts and hydrolysates achieved more than 80% reduction in cell migration, related to the control, and induced a significant increase in cell adhesion after 60 min of treatment. All samples demonstrated a 45–65% reduction in the activity of specific MMPs (MMP-9 and proMMP-9) linked to cancer progression. Cell cycle analysis revealed a remarkable apoptotic effect in cells treated with the PLE hydrolysate. *In vivo* experiments confirmed these results showing that the treatment with hydrolysates resulted in a reduction ranging from 44–63% in tumor volume and from 33–50% in tumor growth. Further steps will focus on the translation of PIs and hydrolysates actives into formulated, deliverable, and *in vivo*–validated drug development programs.

## Supporting information

S1 File Raw data required to replicate all study findings reported in the article. (XLSX)
